# Association between allostatic load and asthma in US adults: A nationwide cross-sectional study

**DOI:** 10.1097/MD.0000000000045326

**Published:** 2025-10-17

**Authors:** Jinzhi Zhang, Wenjie Wang, Qing Miao

**Affiliations:** a Respiratory Department, Xiyuan Hospital, China Academy of Chinese Medical Sciences, Beijing, China; b Capital Medical University, Beijing, China.

**Keywords:** allostatic load, asthma, chronic stress, NHANES, physiological dysregulation, public health

## Abstract

Chronic psychosocial stress has been implicated in asthma pathogenesis, but large-scale population-based evidence remains limited. We examined the association between allostatic load (AL) (a composite marker of multisystem physiological dysregulation induced by chronic stress) and asthma prevalence among U.S. adults using National Health and Nutrition Examination Survey 2005 to 2010 data. The allostatic load index (ALI) was calculated from 8 physiological biomarkers representing cardiovascular, metabolic, and inflammatory systems and was categorized as ≤1, 2, and ≥3 for comparative analysis. Asthma was defined by self-reported physician diagnosis. Survey-weighted logistic regression was used to estimate odds ratios (OR) for asthma across ALI categories, adjusting for age, sex, race/ethnicity, socioeconomic indicators, and smoking. Of 15,533 participants (mean age 45.8 years), 2110 (13.6%) reported a history of asthma. Among these individuals, there were 40.57% ALI ≤ 1, 21.0% ALI = 2, and 38.4% ALI ≥ 3. Each 1-unit increase in ALI was associated with a 13% higher odds of asthma (adjusted OR: 1.13, 95% confidence interval [CI]: 1.08–1.18). Compared to those with ALI ≤ 1, the ALI ≥ 3 group was associated with a 45% higher odds of asthma (OR: 1.45, 95% CI: 1.25–1.69, *P* < .001), with a significant dose–response trend across ALI categories (*P* for trend < .001). In sensitivity analyses applying clinically defined biomarker thresholds, the results remained robust (adjusted OR for ALI ≥ 3 compared with ALI ≤ 1, 1.62; 95% CI, 1.39–1.89). Subgroup analyses revealed significant effect modification by sex and socioeconomic status: the ALI–asthma association was stronger in women and in individuals with poverty-to-income ratio < 1.3 (interaction *P* = .01), but was not statistically significant in men. Associations remained consistent across age, race/ethnicity, smoking status, and education. These findings indicate that cumulative biological stress, as indexed by AL, is associated with asthma in U.S. adults, particularly among women and socioeconomically disadvantaged populations. Incorporating AL into asthma risk assessment may improve identification of high-risk individuals and guide upstream interventions targeting chronic stress. Addressing chronic stress and social determinants of health may be important strategies for reducing asthma burden.

## 1. Introduction

Asthma is a highly prevalent chronic respiratory disease in the United States, affecting an estimated 25 million people (approximately 8% of the population).^[1]^ Despite advances in medical therapies and disease management, asthma continues to exact a heavy toll on health. It accounts for over 1.8 million emergency department visits, roughly 169,000 hospitalizations, and several thousand deaths annually in the U.S. These acute events reflect significant morbidity.^[1]^ Moreover, asthma imposes a large economic burden: total annual costs (including direct medical care and indirect costs such as lost productivity) have been estimated in the 10s of billions of dollars.^[2,3]^ Asthma’s high prevalence, impact on quality of life, and uneven distribution across subpopulations underscore its public health importance.^[2,4]^

Asthma pathogenesis is multifactorial. Established risk factors include genetic predisposition and environmental exposures such as allergens, air pollution, tobacco smoke, and infections.^[5]^ However, accumulating evidence highlights chronic psychosocial stress as an important but often underappreciated contributor to asthma. Persistent stressors (ranging from low socioeconomic circumstances and violence exposure to occupational strain and interpersonal conflict) have been linked to greater asthma incidence and poorer asthma control.^[6]^ These psychosocial adversities disproportionately affect disadvantaged and minority communities that already bear a higher asthma burden. For example, poverty, neighborhood deprivation, and experiences of discrimination are all associated with both elevated asthma prevalence and uncontrolled symptoms.^[6,7]^ In this light, stress is increasingly recognized not merely as a transient trigger of asthma attacks, but potentially as a factor in asthma development and severity.

The construct of allostatic load (AL) offers a framework for quantifying the physiological impact of chronic stress. Allostasis refers to the body’s adaptive process to maintain stability through change, primarily via neuroendocrine, autonomic, and immune responses.^[8,9]^ When stress is chronic or repeated, however, these adaptive systems may become overburdened, leading to cumulative “wear and tear” on multiple organ systems. This cumulative burden is termed AL and is operationalized by composite indices of biological biomarkers.^[10]^ Higher AL has been associated with numerous adverse health outcomes, including cardiovascular disease, metabolic syndrome, diabetes, cognitive decline, mental health disorders, and increased mortality.^[11–13]^ In these contexts, AL integrates diverse signals of physiological dysregulation (such as elevated blood pressure, dyslipidemia, immune activation, and impaired glucose metabolism) reflecting an organism’s cumulative stress burden.

Despite its established link to many chronic diseases, the role of AL in respiratory health has been less explored. There are plausible mechanistic pathways by which chronic stress could promote asthma. Acute stress activates the hypothalamic–pituitary–adrenal axis and the sympathetic–adrenal–medullary system, transiently raising corticosteroids and catecholamines, which can suppress inflammation. In contrast, chronic stress leads to adaptations such as glucocorticoid receptor desensitization and heightened sympathetic tone, resulting in a pro-inflammatory shift and immune dysregulation.^[14,15]^ Over time, sustained stress-induced hormonal changes may favor a T-helper-2 immune profile with increased interleukins (IL-4, IL-5, and IL-13), which underlie allergic inflammation in asthma.^[16]^ Similarly, stress-driven autonomic imbalances can alter airway responsiveness. Collectively, these chronic stress pathways may amplify airway inflammation and hyperresponsiveness, lowering the threshold for allergic reactions and asthma attacks. Emerging experimental and clinical data support this model: for instance, children and adolescents exposed to violence or high stress have higher risk of developing asthma, and laboratory stress exposures can modulate immune markers relevant to asthma.^[17]^

Empirically, research directly linking AL to asthma is sparse. One large community study in the United Kingdom found that adults with asthma had modestly higher AL scores than those without asthma, even after adjusting for medication use.^[18]^ Among younger populations, a study reported that adolescent boys with higher AL had several-fold greater odds of asthma, potentially driven by elevated cardiometabolic markers.^[19]^ These findings suggest that an integrated measure of physiological stress may relate to asthma risk, but data in the general U.S. adult population are lacking.

The National Health and Nutrition Examination Survey (NHANES) is a nationally representative, multiethnic survey of U.S. residents, combining interviews with physical examinations and laboratory tests. NHANES provides comprehensive biomarker data suitable for calculating an ALI. Leveraging NHANES, we conducted a cross-sectional analysis to evaluate the association between AL and prevalent asthma among U.S. adults. Our objective was to determine whether a higher cumulative stress burden, as indexed by AL, is associated with higher asthma prevalence in the general population. We also examined whether this relationship varies across demographic subgroups (e.g., by sex, socioeconomic status) to elucidate potential disparities.

## 2. Methods

### 2.1. Study design and data source

We analyzed data from NHANES cycles 2005 to 2006, 2007 to 2008, and 2009 to 2010. NHANES uses a stratified, multistage probability design to produce a nationally representative sample of the U.S. civilian, noninstitutionalized population. The survey includes household interviews and standardized physical examinations. The study protocol was approved by the National Center for Health Statistics Ethics Review Board, and all participants provided written informed consent. All NHANES procedures received Institutional Review Board approval, and the data are publicly available from the Centers for Disease Control and Prevention.

### 2.2. Study population

**Figure 1.**
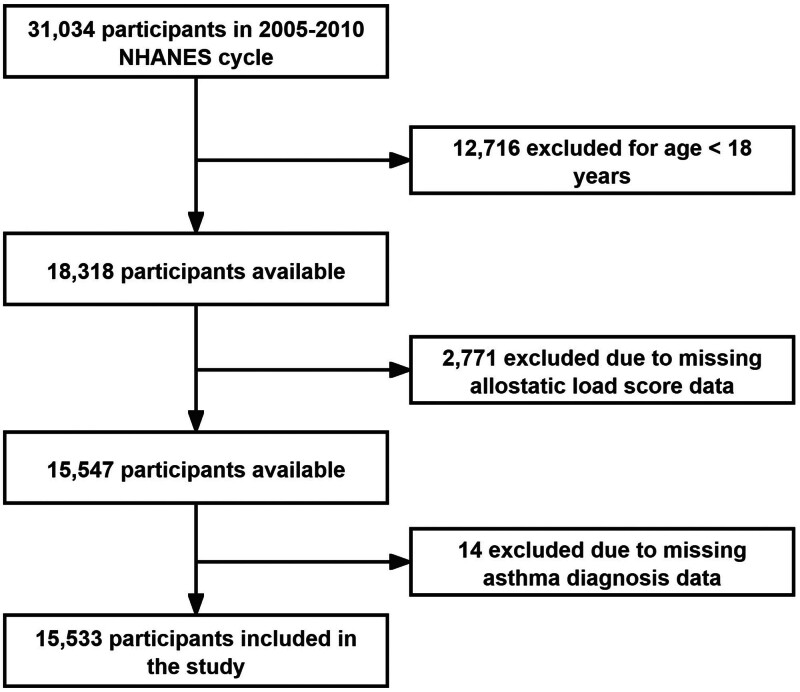
Flow diagram of the screening and enrollment of study participants.

A total of 31,034 participants were surveyed across the NHANES 2005 to 2010 cycles. We applied the following exclusion criteria: excluded individuals younger than 18 years (n = 12,716), because our focus was on adults. We further excluded participants missing data on any of the 8 biomarkers used to calculate the ALI (n = 2771) or missing information on asthma status (n = 14). After exclusions, 15,533 participants (84.8% of the eligible adult sample) remained for analysis (see Fig. 1).

### 2.3. Asthma ascertainment

Asthma status was defined using self-reported physician diagnosis. During the interview, participants were asked: “Has a doctor or other health professional ever told you that you have asthma?” Those who answered “Yes” were classified as having asthma. This case definition is consistent with prior NHANES-based asthma studies.^[20]^ We also considered the presence of asthma-related medication use (such as inhalers) or symptoms (wheezing, coughing) as supportive evidence, but the primary definition was self-reported doctor-diagnosed asthma.

### 2.4. Allostatic load index

The allostatic load index (ALI) was constructed to quantify cumulative physiological dysregulation, following methodologies established in prior NHANES-based studies.^[21,22]^ Consistent with these approaches, 8 biomarkers representing cardiovascular, metabolic, and immune system function were included: systolic blood pressure, diastolic blood pressure, total cholesterol, high-density lipoprotein (HDL) cholesterol, glycated hemoglobin, serum albumin, body mass index (BMI), and C-reactive protein.

For the primary ALI calculation, each biomarker was divided into quartiles based on its weighted distribution in our analytic sample. Participants in the highest quartile were scored as high risk (1 point), except for HDL and albumin, for which the lowest quartile represented high risk. The scores across biomarkers were summed, generating an ALI ranging from 0 to 8. Higher scores reflected greater cumulative dysregulation. For analysis, ALI was categorized as ≤1, 2, or ≥3, consistent with both score distribution and prior literature.^[22]^

In a sensitivity analysis, we alternatively defined risk using established clinical cutpoints: systolic blood pressure ≥ 140 mm Hg, diastolic blood pressure ≥ 90 mm Hg, total cholesterol ≥ 240 mg/dL, HDL < 40 mg/dL, glycated hemoglobin ≥ 6.4%, albumin < 3.8 g/dL, BMI ≥ 30 kg/m², and C-reactive protein ≥ 0.30 mg/dL. Each biomarker meeting the risk threshold contributed 1 point, and the resulting ALI (0–8) was categorized using the same 3-level grouping (≤1, 2, and ≥3). This dual approach ensured that our findings were robust to both distribution-based and clinically defined exposure metrics.

### 2.5. Covariates

We selected covariates based on prior literature on asthma risk and AL determinants. Demographic covariates included age, sex, and race/ethnicity. Age was categorized into 3 groups (18–44, 45–64, and ≥65 years) for descriptive purposes but was treated as a continuous adjustment variable in regression models. Race/ethnicity was self-reported and classified as non-Hispanic White, non-Hispanic Black, Mexican American, or other (including other Hispanic and multiracial). Sex was male or female. Socioeconomic status was approximated by education level and the poverty-to-income ratio (PIR); PIR is the ratio of household income to the poverty threshold, with values < 1 indicating below poverty. We categorized PIR into < 1.3 (low), 1.3 to 3.5 (middle), and ≥3.5 (high). Education was categorized as less than high school (<12th grade), high school/GED (12th grade), or above high school (>12th grade). Marital status was classified as married/living with partner, divorced/separated, widowed, or single (never married). Smoking status was categorized as never smoker, former smoker, or current smoker, based on self-report. These variables were included as potential confounders in adjusted analyses because they may influence both AL and asthma risk.

### 2.6. Statistical analysis

All analyses accounted for the complex sampling design and weights of NHANES, ensuring nationally representative estimates. We used R software (version 4.4.3; R Foundation for Statistical Computing, Vienna, Austria) with the “survey” package to incorporate sampling weights, clustering, and stratification. First, we described the weighted characteristics of the study sample stratified by asthma status. Continuous variables were reported as weighted means with standard errors or medians with interquartile ranges (if skewed), and categorical variables as weighted percentages. We compared differences between participants with and without asthma using chi-square tests for categorical variables and *t* tests or nonparametric tests for continuous variables, as appropriate.

Next, we assessed the association between AL and asthma using weighted logistic regression. Our main exposure was ALI, modeled both as a continuous variable (per 1-unit increase) and categorically. We constructed 3 models to sequentially adjust for potential confounders: Model-1 was unadjusted (crude); Model-2 adjusted for demographic factors (age, sex, race/ethnicity); Model-3 further adjusted for socioeconomic factors (education, marital status, PIR) and smoking status. We report odds ratios (ORs) with 95% confidence intervals (CIs). Trend tests were performed by modeling ALI category as an ordinal variable. The ALI ≤ 1 group served as the reference for categorical analyses. A 2-sided *P* < .05 was considered statistically significant.

We conducted subgroup analyses to examine whether the ALI–asthma association varied across key population groups. Specifically, we stratified models by sex, age group, race/ethnicity, PIR category, smoking status, marital status, and education level. Interaction terms between ALI (continuous) and each subgroup variable were tested. Finally, we reran the regression analyses using the clinically defined ALI measure to confirm consistency of results. Statistical analyses and data visualization were performed using R software (version 4.4.3) with the “survey,” “tableone,” and “ggplot2” packages, along with EmpowerStats software (version 4.2.1; X&Y Solutions, Inc., Boston). *P* values < .05 were deemed statistically significant.

## 3. Results

### 3.1. Characteristics of participants

Among the 15,533 adults included, 2110 (weighted 13.6%) reported a history of physician-diagnosed asthma. The weighted mean age was slightly lower in the asthma group (43.6 years) than in the non-asthma group (46.3 years, *P* < .001), reflecting a higher proportion of participants under 45 among asthmatics. Females were disproportionately represented among those with asthma (57.6% of asthma group vs 50.4% of non-asthma; *P* < .001). Racial/ethnic composition also differed: non-Hispanic Whites comprised a larger share of the asthma group, whereas non-Hispanic Blacks were underrepresented among asthmatics (*P* < .001).

Participants with asthma were less likely to be married or partnered and more likely to be single or divorced (*P* < .01). Smoking prevalence was higher among asthmatics: 26.8% were current smokers compared to 21.5% of those without asthma (*P* < .001). Mean poverty-to-income ratio was lower in the asthma group (2.91 vs 3.07, *P* = .0045), indicating greater economic disadvantage. The asthma group had a higher prevalence of obesity (BMI ≥ 30) (41.7% vs 32.7%, *P* < .001).

Notably, the mean ALI was higher among individuals with asthma (mean ALI 2.00) than among those without asthma (mean ALI 1.81, *P* = .0006). A greater proportion of the asthma group fell into the highest ALI category: 34.5% of asthmatics had ALI ≥ 3 versus 29.8% of non-asthmatics (*P* = .004). These unadjusted comparisons suggest that asthma cases tended to have higher cumulative physiological dysregulation. (Weighted characteristics are summarized in Table 1.)

**Table 1:** Weighted basic characteristics of participants stratified by asthma status.

Characteristics	Total (n = 15,533)	Asthma		*P*
		No (n = 13,423)	Yes (n = 2110)	
Age, mean (SE) (yr)		46.30 (0.37)	43.60 (0.45)	<.0001
Age, No. (%)				.0001
<45		6135 (47.89)	1098 (53.75)	
45–65		4175 (35.30)	633 (32.74)	
≥65		3113 (16.81)	379 (13.51)	
Sex, No. (%)				<.0001
Female		6718 (50.44)	1201 (57.63)	
Male		6705 (49.56)	909 (42.37)	
Ethnicity, No. (%)				<.0001
Non-Hispanic White		6354 (70.21)	1104 (72.77)	
Non-Hispanic Black		2810 (9.24)	226 (4.21)	
Mexican American		2546 (10.21)	481 (12.54)	
Other Hispanic or other Race		1713 (10.35)	299 (10.48)	
Marital status, No. (%)				<.0001
Widowed		1084 (5.95)	139 (4.95)	
Divorced or separated		1681 (11.96)	339 (14.73)	
Married or living as married		7946 (65.58)	1073 (59.18)	
Single		2313 (16.51)	462 (21.14)	
Smoking, No. (%)				<.0001
Past smoker		3169 (24.86)	501 (24.98)	
Current smoker		2667 (21.47)	531 (26.83)	
Never smoked		6780 (53.67)	900 (48.19)	
Education, No. (%)				.2772
<12th grade		4020 (19.37)	557 (17.83)	
12th grade		3267 (24.63)	508 (23.76)	
>12th grade		6118 (56.00)	1044 (58.41)	
PIR, mean (SE)		3.07 (0.04)	2.91 (0.07)	.0045
PIR, No. (%)				.0001
<1.3		3754 (19.04)	719 (24.32)	
1.3–3.5		4767 (36.56)	687 (33.50)	
≥3.5		3864 (44.40)	562 (42.18)	
BMI (kg/m^2^)				<.0001
<18.5		236 (1.83)	34 (1.56)	
18.5–24.99		3937 (31.48)	541 (27.33)	
25–29.99		4633 (34.03)	610 (29.46)	
≥30		4617 (32.67)	925 (41.65)	
ALI, mean (SE)		1.81 (0.02)	2.00 (0.05)	.0006
ALI, No. (%)				.0042
≤1		5921 (48.10)	856 (44.15)	
=2		2997 (22.11)	443 (21.31)	
≥3		4505 (29.79)	811 (34.54)	

Data are shown as n (%).

ALI = allostatic load index, BMI = body mass index, PIR = poverty-to-income ratio, SE = standard error.

### 3.2. Association of AL with asthma

We next examined the multivariable association between ALI and asthma prevalence. In unadjusted logistic regression (Model-1), higher ALI was associated with higher odds of asthma. Treating ALI as a continuous variable, each additional point in ALI corresponded to an 8% increase in the odds of having asthma (OR: 1.08 per unit, 95% CI: 1.04–1.13; *P* < .001). After adjusting for age, sex, and race/ethnicity (Model-2), the effect estimate for continuous ALI increased (adjusted OR: 1.13 per unit, 95% CI: 1.09–1.18; *P* < .001). In the fully adjusted model (Model-3), which included socioeconomic covariates and smoking, the association remained significant (adjusted OR: 1.13, 95% CI: 1.08–1.18; *P* = .001).

When analyzing ALI categorically, we observed a clear dose–response pattern. In the crude model, participants with high ALI (≥3) had 26% higher odds of asthma compared to those with low ALI (≤1) (OR: 1.26, 95% CI: 1.10–1.45; *P* = .002). After full adjustment, high ALI was associated with 45% higher odds of asthma (adjusted OR: 1.45, 95% CI: 1.25–1.69; *P* < .001) (Table 2). The moderate ALI group (ALI = 2) showed a smaller and nonsignificant association in the fully adjusted model (adjusted OR: ~1.16, 95% CI: 0.98–1.39). The trend test across ALI categories was highly significant (*P* < .001).

**Table 2:** The association between ALI and prevalence of asthma (ALI by quartiles).

	Model-1		Model-2		Model-3	
	OR (95% CI)	*P*-val	OR (95% CI)	*P*-val	OR (95% CI)	*P*-val
ALI continuous	1.08 (1.04, 1.13)	<.001	1.13 (1.09, 1.18)	<.001	1.13 (1.08, 1.18)	.001
Quartiles of ALI						
ALI ≤ 1	Ref		Ref		Ref	
ALI = 2	1.05 (0.90,1.22)	.537	1.18 (1.01, 1.37)	.046	1.16 (0.98, 1.39)	.097
ALI ≥ 3	1.26 (1.10,1.45)	.002	1.46 (1.27, 1.67)	<.001	1.45 (1.25, 1.69)	<.001
*P* for trend		<.001		<.001		.001

*Note*: Crude, unadjusted for confounders. Model-1, adjusted for age, sex, race. Model-2, adjusted for model-1 + smoke, marry, PIR, and education.

ALI = allostatic load index, OR = odds ratio.

The sensitivity analysis using clinical biomarker cutoffs yielded similar findings (Table 3). In the fully adjusted model with clinically defined ALI, the high-risk group (clinical ALI ≥ 3) had a 62% higher odds of asthma (adjusted OR: 1.62, 95% CI: 1.39–1.89; *P* < .001) relative to the low-risk group (ALI ≤ 1). The moderate-risk group (ALI = 2) also showed a significant association (adjusted OR: 1.27, 95% CI: 1.10–1.45; *P* = .002). The dose–response trend persisted under this alternative definition (trend *P* < .001).

**Table 3:** The association between ALI and prevalence of asthma (ALI by clinical cut points).

	Model-1		Model-2		Model-3	
	OR (95% CI)	*P*-val	OR (95% CI)	*P*-val	OR (95% CI)	*P*-val
ALI continuous	1.14 (1.08, 1.19)	<.001	1.18 (1.13, 1.24)	<.001	1.19 (1.13, 1.25)	.001
ALI by clinical cut points						
ALI ≤ 1	Ref		Ref		Ref	
ALI = 2	1.15 (1.01, 1.32)	.041	1.25 (1.09, 1.42)	.002	1.27 (1.10, 1.45)	.002
ALI ≥ 3	1.46 (1.26, 1.69)	<.001	1.60 (1.40, 1.84)	<.001	1.62 (1.39, 1.89)	<.001
*P* for trend		<.001		<.001		<.001

*Note*: Crude, unadjusted for confounders. Model-1, adjusted for age, sex, race. Model-2, adjusted for model-1 + smoke, marry, PIR, and education.

ALI = allostatic load index, OR = odds ratio.

### 3.3. Subgroup analyses

**Figure 2.**
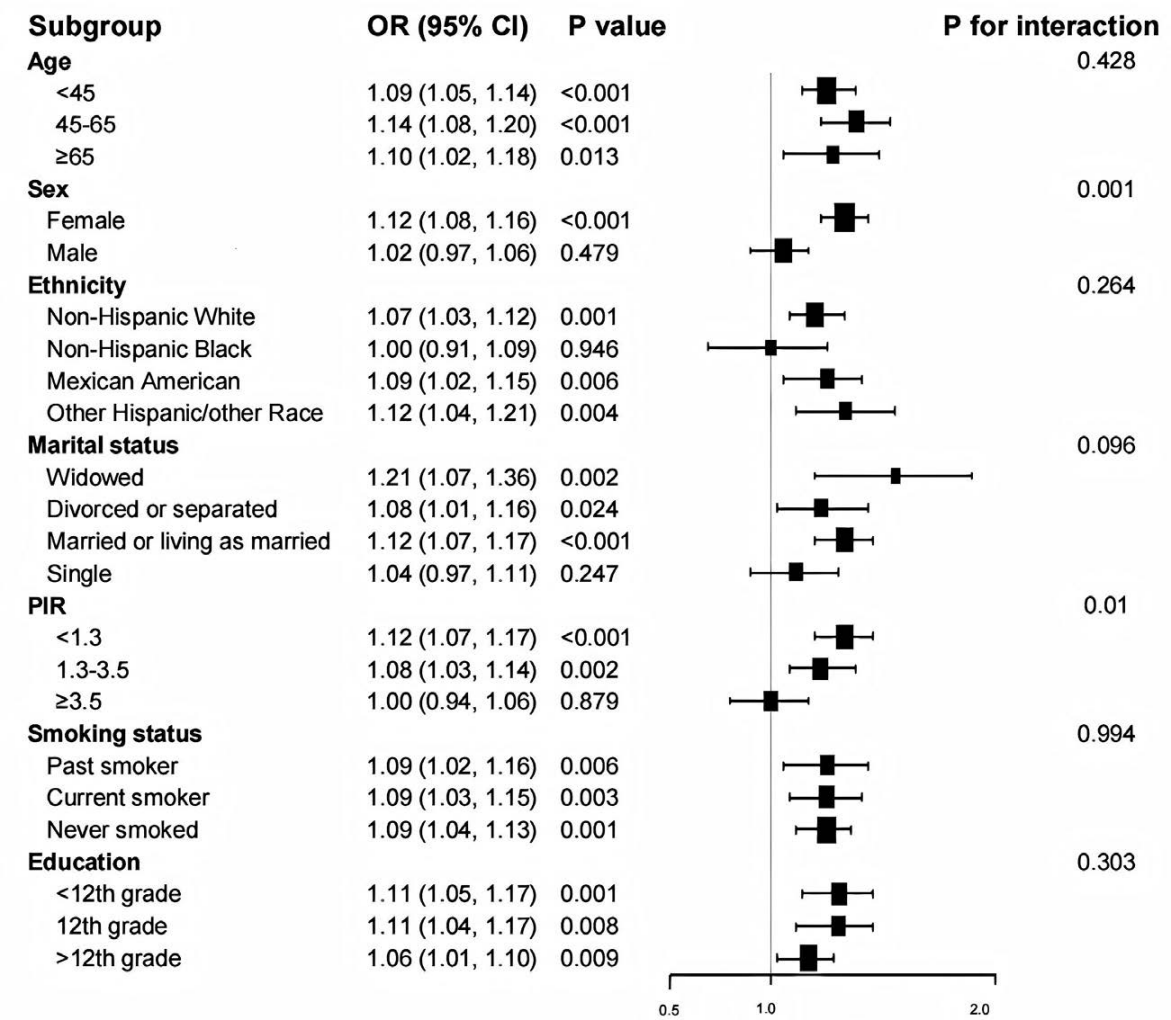
Forest plot between ALI and asthma. ALI = allostatic load index.

To explore potential effect modification, we stratified the analysis by demographic and socioeconomic factors (Fig. 2). The association between ALI and asthma was notably heterogeneous by sex. In women, higher ALI was strongly associated with asthma. In contrast, among men the association between ALI and asthma was not statistically significant after adjustment. The interaction between ALI and sex was significant (*P* for interaction = .001).

We observed a similar pattern across income strata. Among individuals with PIR < 1.3 (the low-income group), higher ALI was significantly associated with asthma (adjusted OR: 1.12 per ALI unit, 95% CI: 1.07–1.17). In contrast, the association attenuated and became nonsignificant in the highest-income group (PIR ≥ 3.5; adjusted OR: ≈1.00, 95% CI: ~0.94–1.06). The interaction by PIR was statistically significant (*P* for interaction = .01), suggesting that the AL–asthma link is more pronounced in socioeconomically disadvantaged populations.

No statistically significant interactions were found for age, race/ethnicity, smoking status, education level, or marital status (all *P* for interaction > .05). The AL–asthma association held consistently across younger and older age groups, across non-Hispanic Whites and Hispanics, and regardless of smoking history. Education and marital status subgroups showed similar trends in AL–asthma association, though CIs were wider.

## 4. Discussion

In this large, nationally representative study of U.S. adults, we found that higher AL was associated with a greater prevalence of self-reported asthma, independent of traditional risk factors. This relationship was monotonic: each unit increase in our ALI conferred higher asthma odds, and those in the highest AL category (ALI ≥ 3) had about 45% higher adjusted odds of asthma than individuals with low AL. Importantly, we observed a clear dose–response trend and consistency across multiple analytic approaches. These results extend the concept of AL to respiratory health, suggesting that cumulative physiological “wear and tear” from chronic stress may be an under-recognized correlate of asthma in adults.

Our findings align with and build upon prior evidence linking stress and asthma. Whereas previous studies in children and specific cohorts implied a stress–asthma link,^[7,15]^ our analysis provides the large-scale population-level confirmation in U.S. adults. For example, a recent UK study reported modestly higher mean AL among adults with asthma compared to controls, supporting the idea that even treated or mild asthma is associated with higher stress burden.^[18]^ By leveraging NHANES data, we demonstrate that this association holds in a multiethnic, community-dwelling sample. The graded relationship we observed is biologically plausible: chronic stressors trigger a cascade of neuroendocrine and immune alterations (e.g., blunted cortisol response, sympathetic overactivity, pro-inflammatory cytokine release) that over time can dysregulate airway physiology.^[7]^ Evidence suggests that stress may alter immune function, potentially leading to the development of asthma or exacerbating its symptoms.^[6]^ However, it remains uncertain whether asthma contributes to elevated AL, or whether high AL predisposes individuals to asthma; a bidirectional relationship mediated by genetic and environmental factors may also be present.

The observed effect modification by sex is noteworthy. Women in our sample exhibited the strongest AL–asthma association, whereas men showed no significant association. This pattern may reflect sex differences in stress physiology and asthma epidemiology. Women generally have higher reported levels of some stress biomarkers and a higher lifetime prevalence of asthma and anxiety disorders.^[16]^ Estrogen and other sex hormones can modulate immune responses, potentially making women more susceptible to stress-related immune shifts. Additionally, psychosocial stressors (e.g., caregiving burden, gender discrimination) may disproportionately affect women. The marked gender disparity in our findings suggests that stress reduction and management interventions might be especially beneficial for women at risk of asthma.

We also found that socioeconomic disadvantage amplified the AL–asthma link. The association between AL and asthma was strongest among participants with low income (PIR < 1.3). This supports the notion that socioeconomic stressors compound biological vulnerability. Low-income individuals often face more persistent stressors (such as financial strain, food insecurity, unsafe neighborhoods, and limited healthcare access) that not only elevate AL but also directly trigger asthma (e.g., through housing conditions or environmental exposures).^[23,24]^ Our findings resonate with life-course models positing that social adversity “gets under the skin” via physiological dysregulation. Allostatic load may therefore serve as a key mediator linking social determinants to asthma outcomes. Interventions aimed at reducing stress and alleviating poverty-related burdens could have a meaningful impact on respiratory health in these high-risk groups.

Several strengths of our study merit emphasis. The use of NHANES ensured a large, diverse, and nationally representative cohort, enhancing the generalizability of our results to U.S. adults. Our ALI was based on objective clinical and laboratory measures from multiple physiological domains, reducing bias from self-report. We adjusted for a comprehensive set of covariates, including demographic, socioeconomic, and behavioral factors, which increases confidence that our findings are not simply due to confounding by factors like age, smoking, or education. The persistence of a dose–response trend across models underscores the robustness of the association. Additionally, sensitivity analyses using clinically defined biomarker cutoffs reproduced the main findings, bolstering the validity of our results.

Nonetheless, our study has limitations inherent to its cross-sectional design. First, temporal ambiguity precludes conclusions about causality. We cannot determine whether high AL preceded asthma onset or whether living with asthma (and its associated stress) elevated AL. Reverse causality is possible: individuals with asthma may experience greater stress (e.g., due to symptom burden or healthcare costs) that contributes to higher AL. However, the observed dose–response pattern and consistency across models lend support to a potentially causal interpretation, which should be confirmed in longitudinal research. Second, asthma status was based on self-report, which may introduce misclassification if participants misunderstood or inaccurately recalled their diagnosis, particularly among older participants. Nevertheless, the NHANES question regarding “doctor-diagnosed asthma” is widely employed in large-scale epidemiological studies, and its reliability has been supported by previous research. Third, our AL measure was based on a single time point. Allostatic load by definition reflects chronic processes, and ideally would be assessed over time. A single measurement may not capture long-term dysregulation or account for acute fluctuations. Finally, although we adjusted for a wide range of covariates, residual and unmeasured confounding cannot be excluded. Unmeasured confounders such as physical activity, environmental exposures, early-life stressors, occupational risks, or genetic predispositions may influence both asthma and AL. Moreover, as our analyses were conducted primarily in U.S. and European populations, the generalizability of our findings to other ethnic and cultural groups is limited. Future multinational, multicenter studies incorporating more diverse populations are needed to strengthen the external validity of these results.

This study does not directly elucidate the mechanism underlying the association between AL and asthma, which requires further validation through clinical and basic experimental studies. Biologically, chronic activation of the hypothalamic–pituitary–adrenal axis and sympathetic nervous system may blunt cortisol responses and promote low-grade inflammation, creating conditions that heighten airway reactivity. Sustained stress can also induce glucocorticoid receptor resistance, weakening endogenous anti-inflammatory control, while prolonged elevations of cortisol and catecholamines may shift immunity toward T-helper 2 responses, profiles commonly observed in allergic asthma.^[16]^ Epigenetic pathways may further mediate this association; pediatric studies have shown that stress and violence exposures are associated with DNA methylation changes in immune-related genes, which in turn correlate with asthma risk.^[23]^ Although these mechanisms remain speculative, it is plausible that cumulative stress imprints on the immune system via epigenetic programming, and AL provides a measurable snapshot of this chronic stress impact.

Our results carry important implications for public health and clinical practice. Recognizing AL as a modifiable risk factor suggests new avenues for asthma prevention. Screening high-risk individuals for elevated AL (using easily obtainable markers like waist circumference, blood pressure, and glycemic control, along with inflammatory markers) could help identify those in whom stress reduction might lower asthma risk. From a social perspective, our findings reinforce the need to address upstream determinants of stress. Policies that reduce socioeconomic hardship, improve neighborhood conditions, and mitigate discrimination could conceivably lower population AL and, in turn, respiratory disease burden. On an individual level, interventions such as cognitive-behavioral therapy, mindfulness, exercise, and social support have been shown to lower stress biomarkers and AL scores.^[24,25]^ Future trials could test whether such interventions reduce asthma incidence or improve control, particularly in vulnerable subgroups identified here. In conclusion, this study provides robust epidemiologic evidence that higher AL is associated with increased asthma prevalence among U.S. adults. The finding of a graded relationship and subgroup disparities enhances its credibility and public health relevance. Our work supports a more holistic, biopsychosocial model of asthma, in which chronic life stress acts alongside allergens and genes to influence disease risk. Addressing the cumulative stress through both clinical and societal measures warrants further investigation as a potential strategy for asthma prevention and reducing health inequities.

## 5. Conclusion

Higher AL (reflecting the cumulative physiological burden of chronic stress) is significantly associated with asthma prevalence among U.S. adults, with evidence of a dose–response relationship. This association appears stronger in women and individuals of lower socioeconomic status. These findings suggest that psychosocial stress and its biological imprint are associated with asthma risk. However, due to the cross-sectional design, causal inference remains limited. Our results underscore the need to broaden asthma prevention strategies beyond traditional allergic and genetic factors to include social and stress-related determinants. In practice, addressing chronic stress through public health initiatives or individual-level stress management could provide novel opportunities to reduce asthma incidence and health disparities. Ultimately, these findings offer a rationale for integrating stress reduction and social equity considerations into asthma research, prevention, and clinical care.

## Author contributions

**Conceptualization:** Qing Miao.

**Data curation:** Jinzhi Zhang, Wenjie Wang.

**Formal analysis:** Jinzhi Zhang.

**Methodology:** Jinzhi Zhang.

**Supervision:** Jinzhi Zhang.

**Writing – original draft:** Jinzhi Zhang, Wenjie Wang, Qing Miao.

**Writing – review & editing:** Qing Miao.
